# Measuring and improving performance of clinicians: an application of patient-based records

**DOI:** 10.1186/s12913-023-09772-2

**Published:** 2023-07-19

**Authors:** Minye Dong, Yuyin Xiao, Chenshu Shi, Guohong Li

**Affiliations:** 1 School of Public Health, Shanghai JiaoTong University School of Medicine, No. 227 South Chonqing Rd, Huangpu District, Shanghai, P.R. China; 2 Center for HTA, China Hospital Development Institute, Shanghai Jiao Tong University, No. 227 South Chonqing Rd, Huangpu District, Shanghai, P. R. China

**Keywords:** Performance measurement, Principal component analysis, Cluster, Benchmark, Difficulty adjustment, Clinician, Human resource management, Electric medical record

## Abstract

**Backgound:**

Efforts to measure performance and identify its driving factors among clinicians are needed for building a high-quality clinician workforce. The availability of data is the most challenging thing. This paper presented a summary performance measure for clinicians and its application on examining factors that influence performance using routine patient-based records.

**Methods:**

Perfomance indicators and difficulty score were extracted from electronic medical records (EMRs). Difficulty adjustment and standardized processing were used to obtain indicators which were comparable between specialties. Principal component analysis (PCA) was used to estimate the summary performance measure. The performance measure was then used to examine the influence of person-job fit and burnout through a mediator effect model and cluster analysis.

**Results:**

A valid sample of 404 clinicians were included in this study, and 244 of them had valid response in the questionnaire. PCA explained 79.37% of the total variance presented by the four adjusted performance indicators. Non-performance attributes and performance driving factors help distinguish different clusters of clinicians. Burnout mediates the relationship between person-job fit and performance in a specific group of clinicians (*β* = 0.120, *p* = 0.008).

**Conclusions:**

We demonstrated the analytical steps to estimate clinicians’ performance and its practical application using EMRs. Our findings provide insight into personnel classified management. Such practice can be applied in countries where electronic medical record systems are relatively less developed to continuously improve the application of performance management.

**Supplementary Information:**

The online version contains supplementary material available at 10.1186/s12913-023-09772-2.

## Introduction

Over ten years have passed since China’s comprehensive health system reform launched in 2009. China has made substantial progress in reducing cost and improving access and quality of healthcare [1–6]. More attention should be paid to the management of human resources, since it is one of three principal health system inputs [7]. It is important for human resources to ensure clinicians are able to maintain their superior status and centrality due to the exclusiveness and specificity of the profession's domain of knowledge [8, 9], and they drive a vast majority of treatment decisions and influence healthcare services [10]. It is estimated that up to 21% of the healthcare costs in the United States can be directly attributed to the services they provide [11]. Clinicians’ high workforce performance is critical to quality service delivery. Hence performance assessments are of great importance.

The most challenging thing in measuring performance is to ensure the results are fair and comparable. Relevant methods have been developed to adjust resource consumption and potential risk, such as Diagnosis Related Groups (DRGs) for hospital payment system and relative value units for medical service pricing and measuring individual labor value [12]. There are also other innovative approaches such as the Indexes of Complexity of Assistance for measuring nursing performance [13], and data envelopment analysis [14]. These methods are always based on a high-quality electronic medical records (EMRs) system and coding system [15]. However, for some countries, use of EMRs is just beginning and systems are not yet standardized. China has pushed the development of EMRs since 2006 [16] and launched a DRG system in 2009. Performance assessments based on these data systems have only been developed for regions, not individuals [17]. Taking into account the practical dilemma of performance assessments in these countries, more explicit and pragmatic methods are needed.

Performance assessments are always used for administrative, feedback and research goals [18], and their ultimate goal is to improve performance [19]. Many factors influence performance of clinicians. In addition to treating patients, clinicians have many other tasks, such as scientific research, teaching, and administrative responsibilities [20, 21]. Between 1998 and 2016, patient visits and inpatient admissions per clinician in China increased by 135% and 184%, respectively [22]. Many Chinese clinicians have also complained about the excessive pressure to publish research [23]. Studies have showed that multi-tasking may hinder performance when individuals’ ability to handle multiple tasks at once does not match the job requirement [24, 25]. These pressures are all factors which may lead to burnout [26, 27]. The overall prevalence of burnout symptoms among clinicians in China ranges from 66.5 to 87.8% [28]. Person-job fit (P-J fit) [29] and burnout [27, 28] both have significant effect on clinicians’ performance. The feeling of being depleted of one’s emotional and physical resources adversely affects the ability to function effectively [30], resulting in a mismatch between individual ability and job demand, which leads to a further decline in performance. Despite many studies examining the relationship between work attitude and subjective performance, little research has used objective performance [31]. A combination of influence factors and objective performance may improve the practical value of performance assessments.

Our study aims to demonstrat the analytical steps to measure clinicians’ performance and further apply the results to identify factors that influence performance. The clinicians in this study mainly refer to doctors who provide clinical services to patients, including internal and surgical medicine. By applying the performance measurement results to daily practice, this study can provide evidence and reference for continuous improvement in the application of performance management. Specifically, we examine the effects of person-job fit and burnout on clinicians’ performance. Four hypotheses are examined.

- Hypothesis 1: High levels of P-J fit are associated with better performance.
- Hypothesis 2: High levels of P-J fit are expected to be associated with low levels of burnout.
- Hypothesis 3: High levels of burnout are linked with poor performance.
- Hypothesis 4: Burnout mediates the relationship between P-J fit and performance.

## Methods

### Study setting and data source

The study sample was 426 clinicians who sought promotion in nine pilot clinical specialties undergoing promotion reform from 67 public hospitals in Shanghai, China, 2020.

Three data sources from Shanghai in 2019 were used: EMRs of inpatient record for performance indicators, personnel files from a human resource information system for basic demographic information, and a survey for attitude to work. The datasets were linked by a unique personal identification number assigned to every clinician.

We adopted some rules for data quality control. In the process of retrieving EMRs, those who had missing key variables or had outliers exceeding five standard deviations on performance indicators were excluded. 404 out of 426 clinicians met the criteria. For the survey, an encrypted link to access the questionnaire was sent to each clinician. A detailed explanation of the scope of the study was provided and informed consent was requested. Responses were uploaded directly to the questionnaire platform, without passing through the clinicians' supervisors. Clinicians who failed to submit questionnaires and whose answer time was too short to be valid were excluded. 254 of 426 clinicians met the criteria. Non-response bias was detected and confirmed to have no effect [32].

### Measurements

#### Developing summary performance measure

In the database of EMRs, each patient can undergo multiple procedures, including surgeries and internal operations. These procedures result in an overall cost and time consumption, and each procedure is performed by one to three clinicians. So, the development of a summary performance score was mainly based on the procedures.

##### Constructing the difficulty score

First, we constructed a mathematical calculation to quantify the difficulty of clinicians’ medical procedure work. Each procedure has been given a grade (1 to 4) based on its technical difficulty, surgical complexity and risk by the health authority, and it is combined into the National Clinical Surgical Operation Classification Code system. For each procedure, a clinician be surgeon, first assistant or second assistant. Each grade and role have been assigned a technical weight by expert opinion. The difficulty score is derived from the grades of the operations a clinician performs and the role in those operations.

In the first sub-step, for a specific clinician $m$ in specific procedure role ${R}_{i}\left(i=\mathrm{1,2},3\right)$, the number of procedures of grade $j$ is ${N}_{mij}\left(j=\mathrm{1,2},\mathrm{3,4}\right)$, and the technical weight (See in Additional file 1: Table A2 and A3) of grade $j$ is ${WG}_{j}\left(j=\mathrm{1,2},\mathrm{3,4}\right)$. Hence, the weighted average of procedure (WAP) for ${R}_{i}$ is given by Eq. (1):

1 $${WAP}_{mi}=\frac{{\sum }_{j=1}^{4}{N}_{mij}*{WG}_{j}}{{\sum }_{j=1}^{4}{N}_{mij}}$$

The second sub-step is to identify the percentage of each role the clinician *m* plays in the procedure. The number of procedures is ${N}_{mi}$. The weighted average percentage of procedure (WAPS) for ${R}_{i}\left(i=\mathrm{1,2},3\right)$ is given by Eq. (2):

2 $${WAPP}_{mi}=\frac{{N}_{mi}*{WAP}_{mi}}{{\sum }_{i=1}^{3}{N}_{mi}}$$

The third sub-step is to calculate the difficulty score. The technical weight (See in Additional file 1: Table A4) of role ${R}_{i}\left(i=\mathrm{1,2},3\right)$ is ${WR}_{i}\left(i=\mathrm{1,2},3\right)$. The difficulty score for clinician *m* is given by Eq. (3):

3 $${DS}_{m}={\sum }_{i=1}^{3}{WAPP}_{mi}*{WR}_{i}$$

According to this definition, the difficulty score is a continuous numerical variable ranging from 0 to 1. The higher the score, the more technically difficult the procedure, the more complex the procedure, and the higher the risk. The difficulty score makes it possible to compare between clinicians. Table 1 presents an example of a single clinician’s difficulty score.

**Table 1:** An example of difficulty score for specific clinician

Operator role	Ni	Procedure grade				WAP	WAPP
		${\mathrm{N}}_{\mathrm{i}1}$: Primary	${\mathrm{N}}_{\mathrm{i}2}$: Secondary	${\mathrm{N}}_{\mathrm{i}3}$: Tertiary	${\mathrm{N}}_{\mathrm{i}4}$: Fourth		
${\mathrm{R}}_{1}$: operator	169	127	37	5	0	0.2834	0.2013
${\mathrm{R}}_{2}$: 1^st^ assistant	65	16	36	12	1	0.4892	0.1336
${\mathrm{R}}_{3}$: 2^nd^ assistant	4	1	2	1	0	0.5000	0.0084

DS = 0.2831

#### Constructing the summary performance score

##### Indicator selection

We selected four indicators under the structure-process-outcome framework [33]. Structure is represented by the annual volume of surgery and operation (VSO). Process is represented by resource consumption, including the average LOS per patient (ALOS) and hospitalization expense per patient (AHE). Outcome is represented by the inpatient mortality rate (MR). We extracted these four indicators from EMRs for the whole population and used the difficulty score to make difficulty adjustment. VSO is multiplied by the difficulty score and the other three indicators are divided by difficulty score. To obtain indicators that are comparable between specialties, we used the deviation from the mean, with maximum difference normalization within specialties. These four adjusted indicators formed the input to principal component analysis (PCA) for constructing the summary performance score.

PCA is a data reduction technique that extracts the effective information from several inter-correlated indicators, and represents it as a set of orthogonal variables called principal components [34]. Mathematically, principal components were obtained from eigen-decomposition of positive semidefinite matrices and upon the singular value decomposition of rectangular matrices.

The principal components were combined into a single measure using the weight calculation method (entropy method in this study [35]). The resulting score was rescaled to a centesimal system by multiplying by 100.

#### Using the performance score to examine P-J fit and Burnout

P-J fit and burnout were measured using standard scales. The score for each scale is calculated by averaging the item score from each question. Both scales met the acceptable threshold (> 0.7) of Cronbach’s α and Kaiser–Meyer–Olkin (KMO) coefficient, with good internal consistency and construct validity [36, 37].

##### P-J fit

P-J fit is attained when an individual’s compatibility with a particular job exists and when an individual possesses the knowledge, skills and attitude which match the job requirements [38]. P-J fit was measured using a 6-item scale developed by Cable and DeRue (2002) (e.g., “The attributes that I look for in a job are fulfilled very well by my present job.”). The responses are anchored on a 5-point Likert scale, ranging from 1 = strongly disagree to 5 = strongly agree.

##### Burnout

Burnout is a psychological stress caused when there is a perceived imbalance between resources and demands, which reduces the motivation and effectiveness of individuals [39] and lead to impaired functioning on the job [40]. We measured burnout with the Chinese version of the 15-item Maslach Burnout Service Inventory (MBI-GS) scale [41] (e.g., “I feel emotionally drained from my work.”). The responses are anchored on a 7-point Likert scale reporting how often a feeling occurs, ranging from 0 = never to 6 = every day. Negative items were scored in reverse.

##### Other variables

We obtained information on clinicians’ gender, education, specialty, management position (including head of department, deputy department director and vice president of hospital) and duration in the current position. For education level, bachelor’s degree served as the reference category. Nine clinical specialties were merged into surgery, internal, gynecology and pediatric, with surgery as the reference category. Duration in the current position was measured in years and treated as a continuous variable. Promotion appraisal results were obtained for verifying the assessment effect. See Additional file 1: Table A1 for variable classification and coding.

### Analytical approach

#### Statistical description and hypothesis testing

Descriptive statistics were calculated for all variables, including demographic characteristics, P-J fit, job burnout and the summary performance score. Bivariate analysis was conducted through one-way non-parametric test and Spearman rank correlation to test the overall differences between groups or variables.

#### Cluster analysis

Unsupervised clustering is a method for discovering groups and identifying new patterns in the data when predefined class labels have not been assigned [42]. Clustering involves partitioning data objects into subsets (clusters) based on similarity or dissimilarity. Samples within a cluster are more similar to each other than to samples in another cluster. We used the K-means algorithm [43] in this study.

#### Mediation analysis

Mediation effect analysis is used to analyze the process and mechanism through which an independent variable influences the dependent variable; that is, how independent variable *X* affects dependent variable *Y* through a mediation variable *M*. The three-step regression procedure introduced by Baron and Kenny was adapted to analyze the mediation effect in order to examine the study hypotheses [44, 45]. The bootstrapping procedure (500 iterations, bias-corrected, 95% Confidence Intervals (95%*CI*)) was applied.

#### Database management and data analysis software

To handle multiple sources of data, DBeaver 4.3.0 database management tool was used to access the database, and filter and link records. SPSS (SPSS for windows 7, version 21.0, SPSS Inc, Chicago, III) and AMOS 24.0 were used for statistical analysis. Visualizations of the results were developed using Medcalc 17.6. The threshold of (two tailed) statistical significance was set at 0.05.

## Result

### Constructing the summary performance score through PCA

**Fig. 1:**
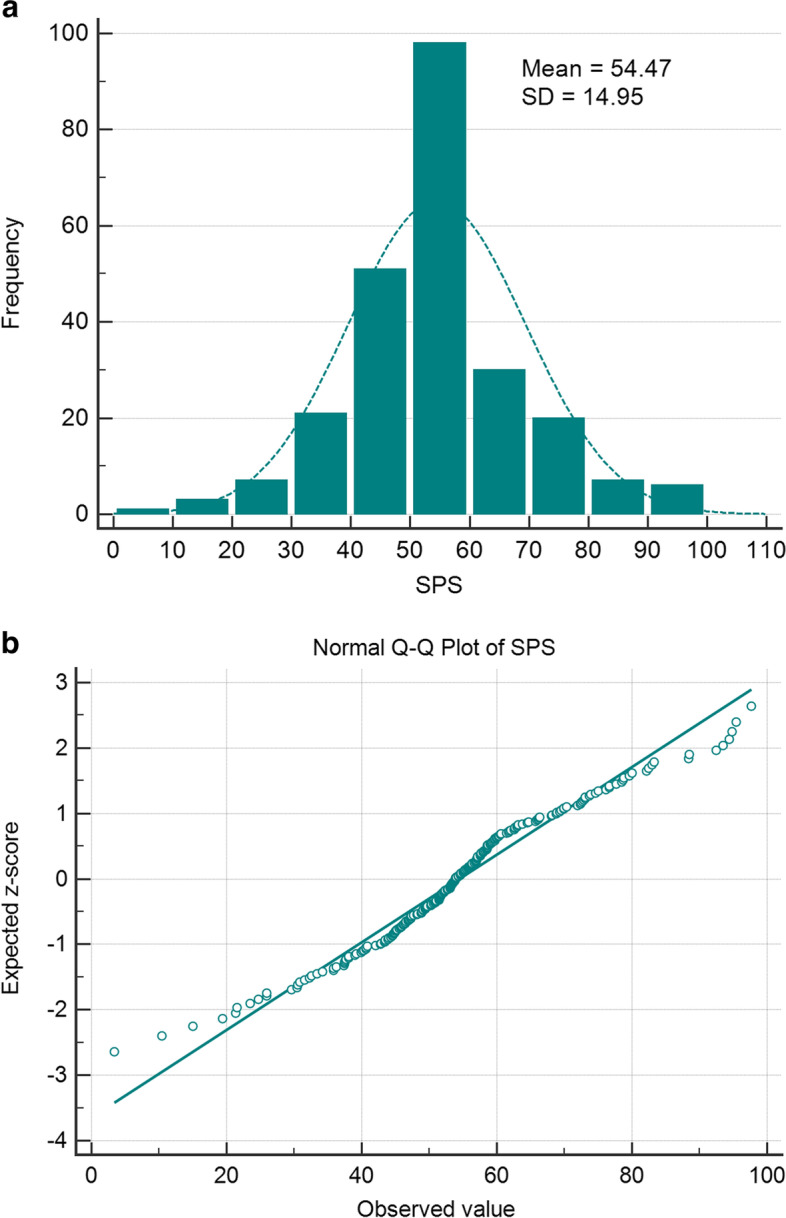
Frequency distribution histogram (**a**) and normal Q-Q plot (**b**) of SPS in total population (*n* = 404)

Three components (F1, F2, F3) were extracted from the PCA, and these explained 79.37% of the total variance presented by the four adjusted indicators. The summary performance score was the weighted sum of these three components rescaled to 0 to 100 (Eq. 4). Figure 1 shows that the summary performance score followed a normal distribution.

4 $$SPS=\left(0.584*F1+0.177*F2+0.240*F3\right)*100$$

Taking appraisal results as a grouping variable, there was no difference between promoted and non-promoted clinicians on the four original performance indicators (p > 0.05). After adjustment, clinicians who were promoted scored higher than those who were not (*Z* = -2.257, *p* = 0.024) (Table 2).

**Table 2:** Descriptive statistical of original performance indicators and SPS (*n* = 244)

Performance indicator	All (*n* = 244) (Mean ± SD)	Appraisal result (Mean ± SD)		*p*
		Not promoted (*n* = 54)	Promoted (*n* = 190)	
VSO	352.44 ± 337.66	269.28 ± 282.78	376.08 ± 348.75	0.061
ALOS	10.77 ± 6.14	11.56 ± 8.39	10.54 ± 5.34	0.637
AHE	37,400.16 ± 28,025.44	33,487.48 ± 27,695.56	38,512.18 ± 28,091.24	0.136
MR	0.01 ± 0.02	0.01 ± 0.02	0.01 ± 0.02	0.533
SPS	54.47 ± 14.95	50.34 ± 13.58	55.65 ± 15.14	0.024

*Note*: The statistical test presented results from non-parametric test since original indicators do not obey normal distribution

### Demographic characteristics

This part of the analysis was conducted in the sample of clinicians who had valid response in both the questionnaire and the summary performance score (*n* = 244). P-J fit, burnout and SPS were significantly different for some of the demographic characteristics (Table 3).

**Table 3:** Summary statistics of variables of valid samples (*n* = 244)

Characteristic		*n*	P-J fit		Burnout		SPS	
			Mean ± SD	*p*	Mean ± SD	*p*	Mean ± SD	*p*
Gender	Female	78	4.08 ± 0.56	0.775	1.36 ± 0.80	0.420	53.23 ± 16.42	0.377
	Male	166	4.05 ± 0.63		1.45 ± 0.80		55.05 ± 14.22	
Age (years)	32–35	17	4.09 ± 0.56	0.951	1.62 ± 0.60	0.509	56.04 ± 14.33	0.936
	36–40	103	4.09 ± 0.57		1.48 ± 0.84		53.92 ± 12.99	
	41–45	75	4.04 ± 0.68		1.36 ± 0.80		55.29 ± 17.02	
	46–50	28	3.99 ± 0.59		1.31 ± 0.88		53.05 ± 16.91	
	51–59	21	4.08 ± 0.54		1.28 ± 0.62		54.93 ± 14.87	
Hospital rating	Secondary	103	3.98 ± 0.49	0.065	1.37 ± 0.77	0.447	50.33 ± 14.69	< 0.001
	Tertiary	141	4.12 ± 0.67		1.45 ± 0.82		57.50 ± 14.44	
Degree	Bachelor	93	3.94 ± 0.61	0.042	1.34 ± 0.82	0.494	51.21 ± 13.81	0.010
	Master	72	4.13 ± 0.57		1.47 ± 0.72		53.05 ± 14.90	
	PhD	79	4.14 ± 0.60		1.46 ± 0.85		59.62 ± 15.09	
Professional title	Attending clinician	186	4.03 ± 0.62	0.185	1.48 ± 0.81	0.030	52.90 ± 13.82	0.003
	Associate chief clinician	58	4.15 ± 0.55		1.22 ± 0.74		59.50 ± 17.28	
Leadership position	No	203	4.07 ± 0.60	0.674	1.44 ± 0.81	0.322	54.79 ± 14.18	0.465
	Yes	41	4.02 ± 0.61		1.31 ± 0.75		52.91 ± 18.38	
Specialty	Surgery	136	4.07 ± 0.64	0.923	1.43 ± 0.81	0.071	55.80 ± 14.39	0.108
	Internal	39	4.03 ± 0.66		1.64 ± 0.80		50.08 ± 15.77	
	Gynecology & pediatrics	69	4.07 ± 0.50		1.27 ± 0.75		54.34 ± 15.28	
Duration in the current position (years)	3–5	14	4.39 ± 0.50	0.009	1.49 ± 0.89	0.973	63.27 ± 14.97	0.037
	6–10	122	4.10 ± 0.57		1.41 ± 0.82		54.98 ± 14.60	
	11–15	83	4.04 ± 0.60		1.40 ± 0.76		53.87 ± 15.22	
	16–23	25	3.75 ± 0.73		1.46 ± 0.84		49.06 ± 14.01	
Appraisal result	Promoted	190	4.07 ± 0.63	0.710	1.43 ± 0.81	0.651	55.65 ± 15.14	0.021
	Not promoted	54	4.03 ± 0.49		1.37 ± 0.78		50.34 ± 13.58	
Summary		244	4.06 ± 0.60	-	1.42 ± 0.80	-	54.47 ± 14.95	-

### Cluster analysis to categorize clinicians

**Fig. 2:**
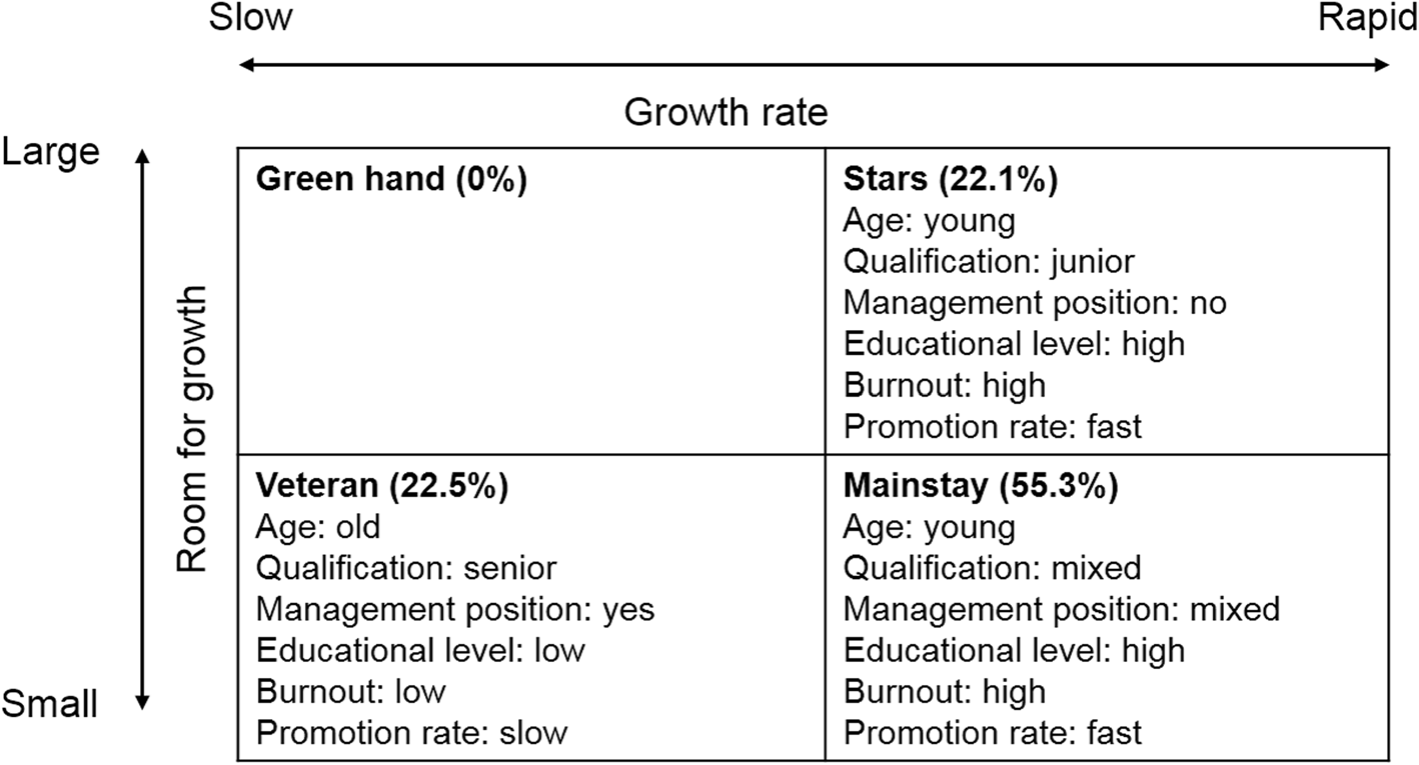
Clusters of the study sample

Using K-means clustering, clinicians were partitioned into three groups based on non-performance attributes (See in Additional file 1: Figure A1). Disparities among clusters were examined for naming the three groups. We used age, whether the clinician holds a management position, professional title and degree to describe the room for growth, and we used degree and duration in the current position to describe the growth rate. Based on these two dimensions, we named the groups as Stars, Mainstays and Veterans (Fig. 2). For more detailed comparison, see Additional file 1: Figure A2, A3 and A4.

### The role of burnout between P-J fit and SPS

P-J fit was positively associated with performance (*β* = 0.175, *p* = 0.006). Burnout was negatively associated with both P-J fit (*β* = -0.560, *p* < 0.001) and performance (*β* = -0.145, *p* = 0.024). Thus, hypotheses *H*_*1*_-*H*_*3*_ were supported (Table 4).

**Table 4:** Means, standard deviations, and correlations of the variables (*n* = 244)

	Mean	SD	P-J fit	Burnout	SPS
P-J fit	4.060	0.603	1	-	-
Burnout	1.418	0.801	-0.560^**^	1	-
SPS	54.473	14.945	0.175^**^	-0.145^*^	1

*Note*: ^*^p < 0.05, ^**^p < 0.01, ^***^p < 0.001

After examining the direct association, we examined the mediator role of burnout between the P-J fit and performance. However, the mediation effect was not supported in all samples. We conducted the analysis in three groups separately and found a significant mediation effect which supported *H*_*4*_ in the group of Stars (Table 5). In the Veteran and Mainstay groups, only the effect between P-J fit and burnout was negatively significant, which supported *H*_*1*_.

**Table 5:** Direct and indirect effects and 95% confidence intervals for mediating models

Grouping	Model pathways	Estimated effect	*p*	95%*CI*	
				Lower *CI*	Upper *CI*
Stars *n* = 54	Direct effect				
	*H*_*1*_: P-J fit → SPS	0.175	0.162	-0.060	0.387
	*H*_*2*_: P-J fit → burnout	-0.383	0.009	-0.579	-0.129
	*H*_*3*_: burnout → SPS	-0.313	0.026	-0.542	-0.056
	Indirect effect				
	*H*_*4*_: P-J fit → burnout → SPS	0.120	0.008	0.038	0.263
Mainstay *n* = 135	Direct effect				
	*H*_*1*_: P-J fit → SPS	0.135	0.327	-0.118	0.353
	*H*_*2*_: P-J fit → burnout	-0.611	0.009	-0.702	-0.458
	*H*_*3*_: burnout → SPS	0.038	0.737	-0.168	0.259
	Indirect effect				
	*H*_*4*_: P-J fit → burnout → SPS	-0.023	0.714	-0.173	0.101
Veteran *n* = 55	Direct effect				
	*H*_*1*_: P-J fit → SPS	0.149	0.308	-0.158	0.443
	*H*_*2*_: P-J fit → burnout	-0.554	0.007	-0.709	-0.324
	*H*_*3*_: burnout → SPS	-0.109	0.448	-0.394	0.153
	Indirect effect				
	*H*_*4*_: P-J fit → burnout → SPS	0.060	0.413	-0.086	0.253

In the absence of mediating variables, the direct effect of the models (the standard regression coefficient between independent and dependent variables) was 0.295 (*p* = 0.025). After introducing burnout, the direct effect of the model decreased to 0.175 (*t* = 1.291, *p* = 0.162). Thus, burnout completely mediated the relationship between P-J fit and performance. The standard regression coefficients between P-J fit and burnout, and between burnout and performance were -0.383 (*t* = -3.015, *p* = 0.009) and -0.313 (*t* = -2.314, *p* = 0.026). The effect size of the mediating effect was 0.120 (*p* = 0.008).

## Discussion

### Performance measure

From four indicators that reflect clinicians’ routine work and could be extracted from EMRs, we developed a difficulty-adjusted performance score. We found no significant difference between promoted and non-promoted clinicians on the original performance indicators, but clinicians who received promotion scored higher on our adjusted score. Many studies aimed at measuring performance or productivity of clinicians focus only on the individual level, which ignores the differences in work content and difficulty [12, 46]. Another study supported that the aggregation of multiple reliable indicators into a composite measure is a useful way to increase the reliability of clinicians’ performance scores [47, 48].

Our performance score uses the same data source for the difficulty score and the performance indicators, and the calculation depends only on the clinicians. Such practices reduce the workload of data collection and avoids involving other data systems like DRGs, so that it was more flexible for countries in similar situations.

### The association of performance measure with burnout and P-J fit

Although composite measures of performance allowed horizontal comparison between clinicians, such practice was of little use on performance improvement. So, we further explored two driving factors of performance: P-J fit and burnout. We grouped clinicians based on non-performance attributes to look for meaningful combinations of clinician features.

Non-performance attributes distinguished Stars and Mainstay from Veterans. We found that Stars and Mainstay had higher burnout and were younger than Veterans. This finding is consistent with a systematic review which found that young clinicians are more at risk of burnout [28]. It explained that young clinicians always served as trainees or junior posts, so they were more likely to be overloaded, work longer hours, and be less rewarded. Furthermore, young clinicians had to follow society's script to complete many of their responsibilities [49], such as marrying and settling down, establishing a circle of friends, taking care of parents and raising children in the context of Chinese traditional culture.

The association of performance score with burnout and P-J fit distinguished Stars from Mainstay and Veteran. Stars had the same level of P-J fit and performance score as the other two groups. Burnout only showed a significant negative effect between P-J fit and performance in Stars. Burnout did hinder Stars’ skills translating to performance. In other words, they should have the potential to achieve better performance if their burnout were alleviated.

Although Mainstays had similar burnout scores as Stars, burnout did not prevent them from using their acquired skills to perform daily tasks. This may be explained by the fact that they were better in resilience. So they could adapt well in face of adversity and stress [50].

Veterans had a lower level of burnout than the other two groups. This may be attributed to the fact that they tend to have senior positions in the organization and have more work experience, which allowed them to better manage their workloads.

### Implications and limitations

It is vital for healthcare human resource managers to understand the distinctions of different clinician segmentation. Our findings can be used to improve performance of clinicians.

Our findings demonstrate that by giving proper attention to the burnout of clinicians like Stars can improve their performance. Such people grow fast and have potential to become outstanding clinicians. However, their emotional processing ability does not match the rapid development of their work, which results in burnout and further hinders performance. Efforts to strengthen resilience and address the pressure from external environments are both effective in reducing burnout [51]. Five options for alleviating burnout have been identified, including staff development, job structure, management development, organizational problem-solving process and agency goals [39]. Hospital administrators could choose and develop appropriate strategies according to the resources and needs of the hospital.

Our findings also recommend differentiating the emphasis of the other two groups. Clinicians like Mainstays are more likely to hold junior positions at this stage. Thus, they may play a limited role in leading clinical decision making. Hospital administrators may benefit from hiring or promoting those employees who can avoid adverse effects of burnout by self-regulation [52]. Proper appointment and issuing clinical privileges may be useful for them to alleviate burnout and enhance self-efficacy [53].

For clinicians like Veterans, although their growth rates are relatively slow, they have become senior employees of the hospital. Their most valuable asset to the hospital is their rich clinical experience. Hospital administrators may take appropriate measures to encourage them to put their practical experience into producing scientific research and improving the quality of medical students.

There are several limitations to this study. First, our inclusion of only four main performance indicators is limited by the availability of data, which may result in the omission of some important but difficult to obtain performance indicators. For example, we were not able to obtain information on patient case mix, which could lead to evaluation bias. Second, since the data source is inpatient records, this can lead to the omission of some important work content of clinicians, such as outpatient work and clinical teaching. Admittedly, the performance indicators we selected in this study could not adapted to all specialists (e.g., most internal medicine physicians). We believe that there is no one-size-fits-all performance measure for all the clinicians, and that it is necessary to adapt the performance evaluation according to the heterogeneity of clinicians' work content. ​Other researchers can select appropriate indicators based on real-world situations in combination with the analysis procedure presented in this study for a comprehensive evaluation, and apply them to practice for performance improvement. In this study, the difficulty scores and performance indicators are derived from the same data source which is in-patient records. For other clinician groups like most internal specialties whose work content is recorded in other data sources, such as out-patient record, indicators used for performance or difficulty measurement need to be further investigated. And corresponding evaluation tools can be continuously designed, implemented, and evaluated in other clinician groups in the further according to the analytical steps and the application presented in this study.

## Conclusion

Our study demonstrates the analytical steps to measure clinicians’ performance and its practical application based on patient-based records. We applied difficulty adjustment to ensure the rationality of the evaluation results. Using composite scores, we further examine the driving factors of performance. The resulting clusters provide insight into personnel management. Such practice can be relatively applied in countries where electronic medical record systems are relatively less developed since similar data sources are available in most areas.

## Supplementary Information

**Additional file 1:** **Table A1. **Definitions of clinician level variables. **Table A2.** Part of fact table of procedure level. **Table A3.** Fact table of proceduregrading. **Table A4.** Fact table of procedure role. **Figure A1.** Dendrogram ofClustering. **Figure A2.** Distribution of continuous demographic characteristic by clusters (*n*=244). **Fig. A3.** Distribution of classified demographic characteristic by clusters (*n*=244). **Fig. A4.** Distribution of SPS by clusters (*n* =244).

## Data Availability

The datasets generated and/or analyzed during the current study are not publicly available due to personal privacy and interests but are available from the corresponding author on reasonable request.

## Abbreviations

EMRs: Electric medical records
P-J fit: Person-environment fit
PCA: Principle component analysis
DRGs: Diagnosis related groups
WAP: Weighted average of procedure
WAPP: Weighted average percentage of procedure
VSO: Annual volume of surgery and operation
ALOS: Average length of stay per patient
AHE: Hospitalization expense per patient
MR: Inpatient mortality rate
KMO: Kaiser-Meyer-Olkin
SPS: Summary performance score
CI: Confidence intervals
