# Supplementary material for: Measuring and improving performance of clinicians: an application of patient-based records
Source: BMC Health Serv Res. 2023 Jul 19;23:775. doi: 10.1186/s12913-023-09772-2 (PMC10357785; doi:10.1186/s12913-023-09772-2)
Supplement: Supplementary file 1 — Additional file 1: Table A1. Definitions of clinician level variables. Table A2. Part of fact table of procedure level. Table A3. Fact table of proceduregrading. Table A4. Fact table of procedure role. Figure A1. Dendrogram ofClustering. Figure A2. Distribution of continuous demographic characteristic by clusters (n=244). Fig. A3. Distribution of classified demographic characteristic by clusters (n=244). Fig. A4. Distribution of SPS by clusters (n =244). [file 12913_2023_9772_MOESM1_ESM.docx]

**Additional files**

**Table A1 Definitions of clinician level variables**

| Variable | Definition and explanation |
| --- | --- |
| Age |  |
| Gender | 1=male, 2=female |
| Degree | 1=bachelor, 2=master, 3=doctor |
| Professional title | 1=attending clinician, 2=associate chief clinician |
| Specialty | 1=surgery, 2=internal, 3=gynecology & pediatric |
| Hospital rating | 1=secondary, 2=tertiary |
| Leadership position | 1=yes, 2=no |
| Duration in the current position | Years of experience in current position |

**Table A2 Part of fact table of** procedure **level**

| Procedure code | Procedure name | Procedure grading |
| --- | --- | --- |
| 0.94001 | Intraoperative neurophysiological monitoring | Primary |
| 1.01002 | Ventriculocardiocentesis with transventricular shunt catheter | Secondary |
| 0.61013 | Percutaneous carotid balloon dilatation | Tertiary |
| 0.50001 | Biventricular pacemaker implantation | Quartus |
| …… | …… | …… |

**Table A3 fact table of** procedure **grading**

| Grading id | Procedure grading | Grading difficulty degree |
| --- | --- | --- |
| 1 | Primary | 0.2 |
| 2 | Secondary | 0.5 |
| 3 | Tertiary | 0.8 |
| 4 | Quartus | 1.0 |

**Table A4 Fact table of** procedure **role**

| Role id | Procedure role | Role difficulty degree |
| --- | --- | --- |
| 1 | Operator | 1 |
| 2 | First assistant | 0.6 |
| 3 | Second assistant | 0.2 |

Figure A1 presented the dendrogram of the clustering. It shows from left to right how clinicians are grouped. At first each clinician was grouped by himself, then the nearest two were combined until all the samples were grouped. The horizontal axis represents the decision value of the combination between clusters, that is, the relative distance between clusters. The relative distance variation can be used to judge the clustering effect. There were two alternative grouping plans according to the figure. Plan A divided the sample into three clusters, among which cluster 1 and cluster 2 were much similar with each other, so they were combined into one cluster in Plan B. In accordance with the principle of interpretability, clinicians were divided into three clusters (Plan A) in this study.


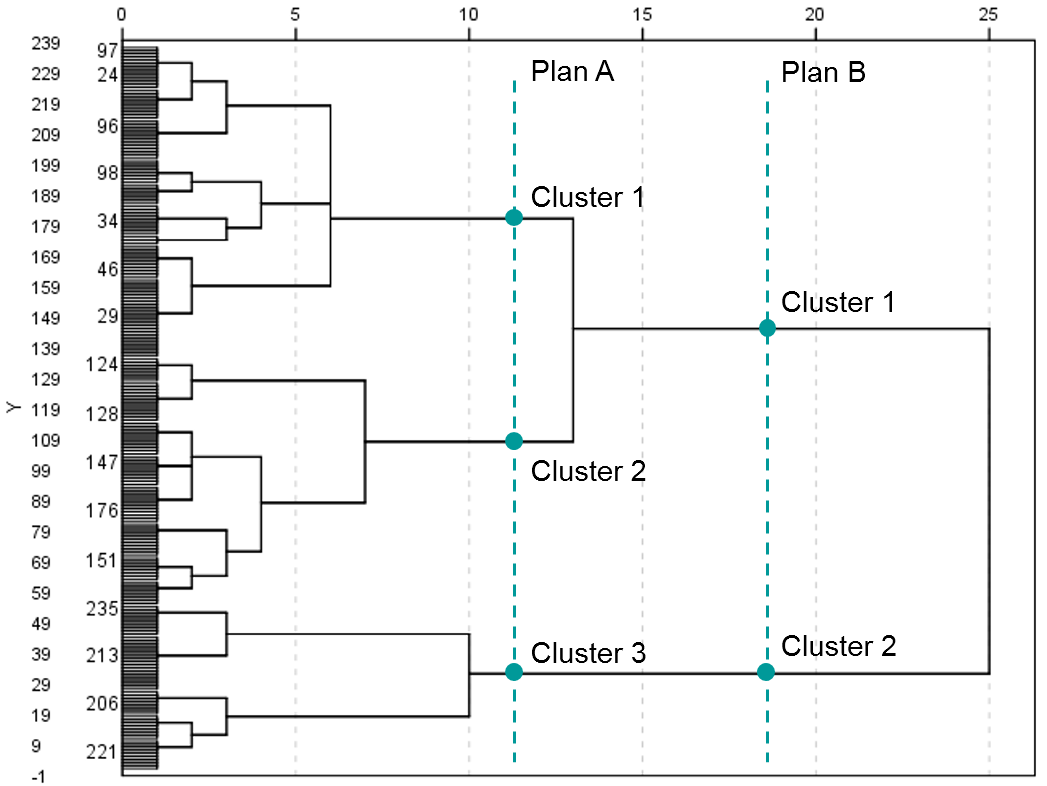


Figure A1 Dendrogram of Clustering

We used the box diagram to show the distribution of continuous variables and percentage bar chart for classified variables of three clusters (Figure A2 and A3). Figure A4 showed that there is no significant difference in performance levels between the three clusters.

Figure 2b showed that the Mainstay and Stars took less time to get promoted than the Veterans. Figure 2a and 4d showed that the Mainstay and Stars are younger while they tend to have a higher burnout level than Veterans. Figure 3d and 3e showed that the Stars have a larger proportion have no management positions and take on junior professional titles than the other two groups. And as showed in figure 3c, they had higher levels of education, which means they had laid a solid foundation for their medical career and were potential for development.


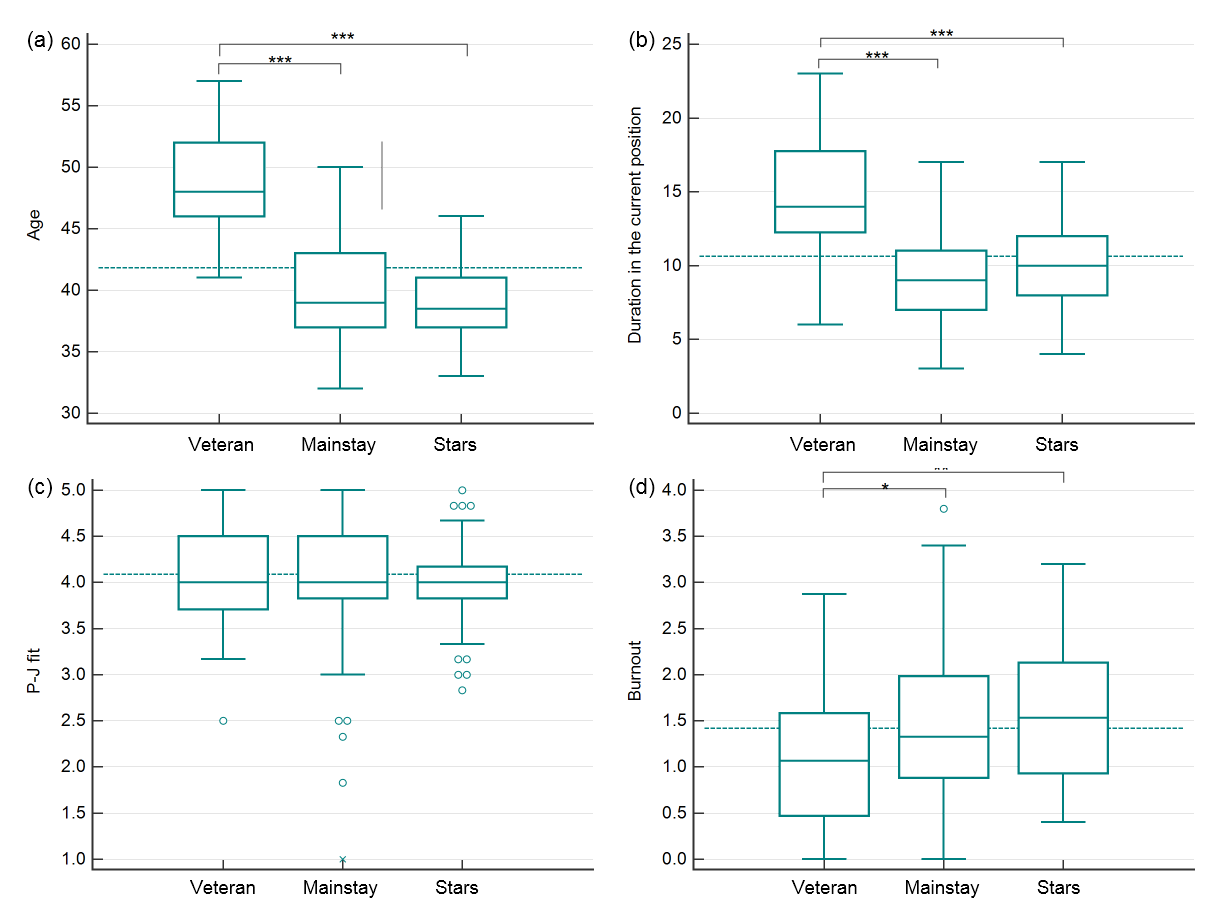


Note: ^*^p<0.05, ^**^p<0.01, ^***^p<0.001

Figure A2 Distribution of continuous demographic characteristic by clusters (*n*=244)


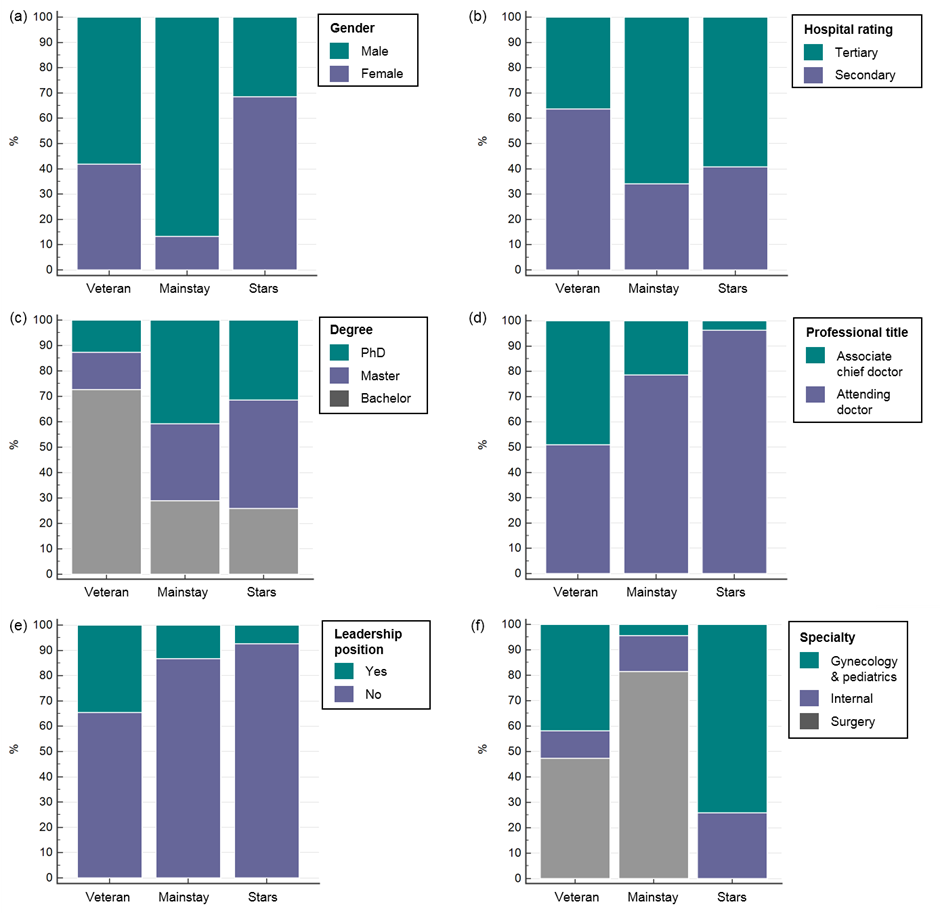


Fig A3 Distribution of classified demographic characteristic by clusters (*n*=244)


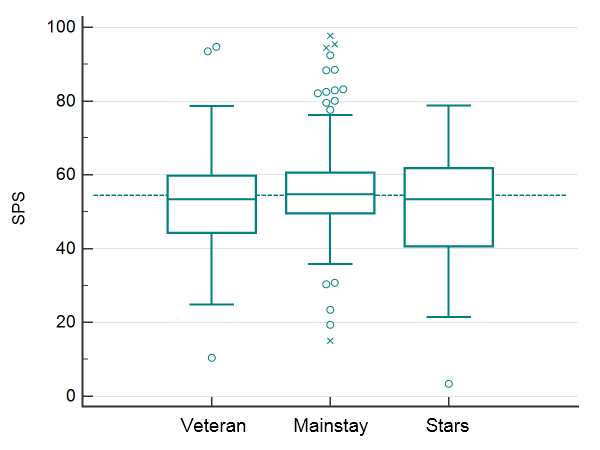


Fig A4 Distribution of SPS by clusters (n =244)
